# Toward a Culturally Situated SDT–EI Model of emotional intelligence in physical education: a narrative review

**DOI:** 10.3389/fpsyg.2026.1914996

**Published:** 2026-08-12

**Authors:** Jiayin Lin, Huanling Ma

**Affiliations:** Guangxi Normal University, Guilin, China

**Keywords:** basic psychological needs, cross-cultural comparison, emotional intelligence, narrative review, physical education, self-determination theory, sport pedagogy, teacher emotional labor

## Abstract

Emotional intelligence (EI) has emerged as a pivotal construct in educational psychology; however, understanding of how physical education (PE) contributes to its development remains fragmented. Eight prior systematic reviews have concluded that PE can develop EI, but three gaps persist: no integration across educational stages, no unified mechanistic account, and no cross-cultural perspective—despite emerging evidence that EI pathways differ across cultural contexts. This narrative review synthesizes 64 publications—including primary studies, eight systematic reviews, and meta-analyses published from 2014 to 2026, together with foundational theoretical works outside that window—through thematic synthesis complemented by an exploratory bibliometric scan, effect direction plotting, and transparent evidence grading, organized around self-determination theory (SDT) as a unifying mechanistic framework with culture positioned as a formal boundary condition. The review centers on PE as a curricular subject in formal educational settings; organized sport enters it only where embedded in educational programs. Four pedagogical models are identified—Teaching Games for Understanding combined with Sport Education, the Sport Education Model, cooperative learning and Teaching Personal and Social Responsibility, and mindfulness/body-expression approaches—and their shared effectiveness is best accounted for by their convergent capacity to satisfy basic psychological needs. The Mercader-Rubio research program and independent cross-validations are synthesized into an SDT-based mechanistic account whose supporting evidence is predominantly cross-sectional and self-report-based and therefore associational rather than causal. Drawing on three direct cross-cultural comparisons, evidence is presented that multiple key links in this chain are culturally modulated. These findings are integrated into a proposed Culturally Situated SDT–EI Model of Physical Education—a theoretically derived framework that has not yet been tested as an integrated structure—generating three design principles for culturally responsive PE–EI programs and eight priorities for the empirical validation the model now requires.

## 1 Introduction

Emotional intelligence (EI)—the capacity to perceive, understand, manage, and utilize emotions (Salovey and Mayer, 1990)—has become a central construct in educational psychology. Meta-analytic evidence confirms that EI predicts academic performance beyond cognitive ability and personality (MacCann et al., 2020), with a meta-analytic correlation of *ρ* = 0.20 (k = 1,246, *N* = 42,529), and school-based social–emotional learning programs produce significant improvements in emotional skills (Cipriano et al., 2023), in a meta-analysis synthesizing 424 studies and 575,361 students. The scientific question is no longer whether EI matters, but how it develops—through what experiences, in what contexts, by what mechanisms, and for whom.

Physical education (PE) occupies a distinctive and undertheorized position within this broader landscape. Unlike classroom-based SEL, which typically addresses emotional competence through verbal instruction, role play, and cognitive reflection, PE engages the whole person in motion. It requires students to manage frustration after errors, regulate arousal under competitive pressure, coordinate with teammates under time constraints, and experience the emotional consequences of their physical actions in real time. These demands—simultaneously physical, cognitive, social, and emotional—make PE a uniquely intensive context for EI development (Rico-González, 2023). A missed penalty kick is not a hypothetical scenario discussed in a workbook; it is a publicly witnessed failure whose emotional residue must be managed before the next play.

### 1.1 What prior reviews have established—and three persistent gaps

Eight systematic reviews published between 2019 and 2025 have mapped the PE–EI terrain (Ubago-Jiménez et al., 2019; Fernández-Espínola and Almagro, 2019; Gil-Moreno and Rico-González, 2023; Rico-González, 2023; Baena-Extremera et al., 2025; Murillo-Pulido et al., 2025; Rivas-Valenzuela et al., 2025; Zhou et al., 2025). Collectively, they conclude that PE can develop EI, that pedagogical design matters, and that motivation—particularly intrinsic and self-determined forms—plays a central role. Table 1 summarizes their contributions and silences.

**Table 1:** Prior systematic reviews on EI in physical education and their unaddressed gaps.

Review	Studies (n)	Focus	Key finding	Gap unaddressed
Ubago-Jiménez et al. (2019)	24	Sport/PE broadly	EI improves sport competencies; educational settings predominate	No mechanism; no cultural analysis
Fernández-Espínola and Almagro (2019)	6	Motivation–EI links	Task orientation and self-determined motivation positively associated with EI	Only 6 studies; no mechanism
Gil-Moreno and Rico-González (2023)	6	Preschool PE	Increasing PA positively influences emotional competence	Preschool only
Rico-González (2023)	28	General PE	Four effective program types; consistent EI–motivation links	No cross-model comparison; no cultural analysis
Baena-Extremera et al. (2025)	10	Primary PE	Most effective didactic strategies identified; continuous teacher training essential	Primary only
Murillo-Pulido et al. (2025)	11	Primary PE	Integrated movement + emotion approaches produce more lasting effects than traditional PE	Primary only
Rivas-Valenzuela et al. (2025)	9	Primary–Secondary	Systematic emotional skills models in teacher training critical; sport improves emotional balance	No mechanism; no cultural comparison
Zhou et al. (2025)	105	SDT in PE (bibliometric)	Intrinsic motivation predicts motor skills, PA, and cognitive learning; cultural moderation under-researched	SDT-focused; EI not primary outcome

Three systematic gaps emerge. First, no integration across educational stages—each review is bounded by a specific age range despite EI development being cumulative and stage-sensitive (Mayer and Salovey, 1997). Second, no mechanistic unification—reviews identify that certain models work but none organize findings into a formal mechanistic account, despite SDT offering the most coherent candidate framework (Ryan and Deci, 2000). Third, no cross-cultural perspective—every review draws predominantly from Western samples, despite emerging evidence that EI pathways differ across cultures (Rogaleva et al., 2024; Zayed et al., 2025) and despite the persistent WEIRD bias in sport psychology (Cormack and Hand, 2022).

### 1.2 Aim and scope

This narrative review aims to synthesize the evidence into a proposed Culturally Situated SDT–EI Model of Physical Education—an integrative framework specifying (a) pedagogical antecedents, (b) SDT-based psychological mechanisms, and (c) cultural boundary conditions. The model is advanced as a theoretically derived, falsifiable proposition generated by the synthesis; it is not an empirically validated structure, and Sections 7.1 and 7.3 specify the evidence that would be required to test it. The review encompasses empirical research, systematic reviews, and meta-analyses published between 2014 and 2026 spanning preschool through university, with foundational theoretical works exempt from the time restriction. Its unit of analysis is physical education as a curricular subject in formal educational settings; organized and competitive sport enters the review only where embedded in educational programs—school sport seasons, university sport science curricula, and student-athletes enrolled in such programs—and the elite-performance sport literature lies outside its scope.

The remainder of the review is organized as follows. Section 2 details the methodological approach. Section 3 establishes the conceptual and measurement foundations. Section 4 examines pedagogical models as antecedents. Section 5 develops the SDT-based mechanistic account. Section 6 introduces culture as a formal moderator. Section 7 synthesizes these layers into the integrated model, articulates its contributions, and identifies its boundary conditions and the research agenda they imply.

## 2 Methods

### 2.1 Review type and rationale

This article employs a narrative review methodology, selected for epistemological fit rather than convenience. The review’s aim—to construct an integrative framework spanning pedagogical antecedents, psychological mechanisms, and cultural boundary conditions—requires a mode of synthesis able to draw connections across heterogeneous study designs, theoretical traditions, and cultural contexts. The governing question is generative rather than evaluative: not how large the effect of PE on EI is, but through what mechanisms and under what cultural conditions PE develops EI. Questions of the first kind are best answered by systematic review and meta-analysis, which derive their authority from an exhaustive, protocol-bound search and a pooled effect estimate. Questions of the second kind require the interpretive latitude to place a cross-sectional Spanish study of need satisfaction alongside a Tunisian scale validation and a Chinese–Russian comparison and to ask what the three jointly imply about a mechanism none of them was designed to test. A meta-analysis, however, desirable for circumscribed questions of intervention effectiveness, is ill-suited to a theory-building task of this scope; with six intervention studies employing heterogeneous EI instruments across only three countries, pooling would in any case violate the homogeneity assumptions on which a defensible pooled estimate depends.

Because the review borrows several procedures conventionally associated with systematic reviews, its departures from that tradition are stated explicitly rather than left to inference. This review is not PRISMA-compliant and should not be read or cited as a systematic review. Five differences are consequential. First, no protocol was registered *a priori* with PROSPERO or an equivalent registry, so the search and the synthesis were refined iteratively as the conceptual structure emerged, whereas a systematic review fixes both in advance. Second, screening was conducted collaboratively to consensus rather than through dual independent screening with a reported inter-rater agreement statistic; the record counts in Section 2.4 are accordingly approximate indications of the screening effort rather than the exact, reproducible figures a PRISMA flow diagram requires. Third, no formal risk-of-bias instrument was applied—neither RoB 2 or ROBINS-I for the primary studies nor AMSTAR 2 for the included reviews. Study quality entered the synthesis instead through the reporting-adequacy criterion in Table 2 and through the evidence-grading scheme in Section 2.6, which grade the strength of a claim across studies rather than the internal validity of each study individually. Fourth, inclusion was partly purposive: Foundational theoretical, measurement, and cultural works were admitted outside the search window because the framework could not be constructed without them, whereas systematic review inclusion is determined solely by protocol-specified eligibility. Fifth, no effect sizes were pooled, and no formal certainty assessment such as GRADE was undertaken; the effect direction plot in Section 2.7 is a structured display of the direction of findings, not an estimate of their magnitude.

**Table 2:** Inclusion and exclusion criteria.

Dimension	Inclusion	Exclusion
Topic	EI or emotional competence in PE, sport education, or organized physical activity in educational settings	Elite/professional sport performance with no educational context; clinical interventions with no PE component
Population	Students from preschool to university; PE teachers; student-athletes in educational programs	Professional athletes outside educational programs; clinical populations without a PE context
Study design	Empirical research (RCT, quasi-experimental, cross-sectional, longitudinal, qualitative); systematic reviews; meta-analyses; conceptual analyses; foundational book chapters	Conference abstracts; unpublished dissertations; non-peer-reviewed preprints
Language	English; Spanish (with translation support)	Other languages (beyond the screening and full-text capacity of the review team; see Sections 2.2 and 2.9)
Publication type	Peer-reviewed journal articles; edited book chapters (theoretical foundations)	Non-peer-reviewed reports; institutional publications
Timeframe	2014–2026 for empirical work; no restriction for theoretical foundations	—
Reporting quality	Adequate reporting of sample, measures, and analyses	Insufficient methodological detail to evaluate findings

What the review does borrow from the systematic tradition—a documented search strategy, explicit inclusion criteria, systematic coding, declared analytic lenses, and transparent evidence grading—is borrowed for a specific and limited purpose: to make the evidence base traceable and the analytic decisions auditable, so that a reader can locate the studies behind each claim and judge the grade assigned to it. These are transparency mechanisms, not claims to exhaustiveness; methodological transparency does not require methodological uniformity, and adopting the former does not entail the latter. The trade-off is deliberate and worth stating plainly. The narrative approach buys conceptual reach—the capacity to integrate across educational stages, mechanisms, and cultures—at the cost of reproducibility and at the risk of selective emphasis. Three safeguards were adopted to contain that risk: The analytic lenses are declared rather than assumed (Section 2.8), non-significant and unmeasured outcomes are made visible rather than silently omitted (Section 2.7), and every pathway in the resulting model carries an explicit evidence grade (Table 3), so that readers can see which claims rest on meta-analytic support and which rest on a single study.

**Table 3:** Evidence direction map: SDT–EI pathways in physical education.

Pathway	Evidence grade	Key caveat
Teacher autonomy support → Student EI	▲▲	Student EI measured as mediator; direct link underexplored
EI → Autonomy need satisfaction	▲	Single cross-sectional Spanish sample; no independent replication
EI → Competence need satisfaction	▲	Direct evidence from one Spanish sample; Turkish self-efficacy findings provide indirect conceptual support, not a replication of competence-need satisfaction
EI → Relatedness need satisfaction	?	Null finding in primary test; may reflect measurement or cultural factors
Need satisfaction → Autonomous motivation	▲▲▲	Meta-analytic support across multiple PE contexts
Pedagogical model → Need satisfaction → EI	▲	No formal mediation analysis; inferred from parallel improvements
Cultural modulation of pathways	▲▲	Three direct comparisons plus one single-country validation; all show culturally specific pathway parameters
Teacher emotional labor → Implementation fidelity → Student EI	?	Untested; teacher-side and student-side literatures have not been linked in a single design

Grades index the consistency and design strength of the evidence supporting each link, not the magnitude of the association. Because EI, need satisfaction, and motivation are measured by self-report in nearly every study contributing to these grades, the grades for links involving EI should be read as upper bounds on the strength of the underlying mechanistic relation (Section 3.2). No entry in this table should be read as a causal estimate (Section 5.3). Evidence grades: ▲▲▲ = consistent evidence with meta-analytic support; ▲▲ = consistent evidence from two or more studies; ▲ = single-study or mixed evidence;? = insufficient or contradictory evidence.

### 2.2 Search strategy

A comprehensive search was conducted across six electronic databases—Web of Science Core Collection, Scopus, PubMed, ERIC, SPORTDiscus, and Google Scholar (the last for gray literature and supplementary verification)—covering January 2014 to June 2026, with foundational theoretical works (e.g., Salovey and Mayer, 1990; Markus and Kitayama, 1991; Ryan and Deci, 2000; Petrides and Furnham, 2001) exempt from the date restriction. The Boolean strategy combined four thematic blocks, adapted to each database’s controlled vocabulary where available: (1) emotional-intelligence terms (“emotional intelligence,” “emotional competence,” “trait/ability emotional intelligence,” “emotional skills,” “emotion regulation”); (2) physical-education and sport terms (“physical education,” “sport education,” “sport pedagogy,” “physical activity,” “athlete”); (3) mechanism and context terms (“self-determination theory,” “basic psychological needs,” “motivation,” “autonomy support,” “cross-cultural,” “collectivism,” “individualism,” “WEIRD”); and (4) educational-context terms (“student,” “teacher,” “school,” “university,” “primary/secondary/higher education”). The core search paired Block 1 with Block 2; mechanism and context searches added Blocks 3 and 4, respectively, to locate studies at the intersection of EI, PE, and the specific mediator and moderator variables of theoretical interest. Search strings were executed in English, with Spanish-language records retrieved through the same international databases and screened with translation support. No searches were run in Chinese, Arabic, Portuguese, French, or other languages, and no regional or national database was interrogated—no SciELO or Redalyc for Latin America and the Iberian Peninsula, no CNKI or Wanfang for China, no CiNii or J-STAGE for Japan, no KCI or RISS for Korea, no African Journals Online for sub-Saharan Africa, and no IndMED or Shodhganga for South Asia. This was a scoping decision taken to keep the corpus within the screening capacity of the review team in the languages available to it; its consequences for the reach of the resulting model are analyzed in Section 2.9 and revisited in Section 7.3.

### 2.3 Inclusion and exclusion criteria

Table 2 specifies the criteria applied during screening.

### 2.4 Screening and selection

The search identified approximately 600 records. After automated and manual deduplication, approximately 400 unique records remained. Title-and-abstract screening removed records that did not meet the inclusion criteria—primarily elite-sport-performance studies, clinical interventions, and nursing-education studies without a PE component—leaving approximately 120 articles for full-text assessment. Full-text review excluded further studies for insufficient methodological detail, populations outside educational settings, topics not directly addressing EI in PE, or unavailable full text. The resulting evidence base comprises the 64 publications cited in this review, encompassing the empirical corpus together with the foundational theoretical, methodological, and cross-cultural works that inform the synthesis. Where eligibility was ambiguous, two authors discussed the study to consensus. Record counts are reported as approximations because the search was refined iteratively rather than executed once against a fixed protocol; they are given to convey the scale of the screening effort and are not offered as the exact figures a PRISMA flow diagram would require. Consistent with the rationale set out in Section 2.1, the review was not registered and does not claim PRISMA compliance; it follows instead a logic of transparent, documented decision-making appropriate to a narrative synthesis.

### 2.5 Bibliometric orientation

To orient the synthesis toward the structure of the literature rather than impose a structure upon it, an exploratory keyword co-occurrence scan of the corpus was performed using VOSviewer (version 1.6.20), retaining keywords that appeared in at least two publications. This scan was used only to surface candidate thematic groupings that informed—rather than determined—the identification of the four pedagogical models and the review’s overall organization. It is reported here as a methodological aid; a formal co-occurrence or citation-burst analysis, and the visualizations such an analysis would produce, lay beyond the scope of this narrative synthesis, and are not presented as stand-alone results.

### 2.6 Thematic synthesis

The primary analytical method was thematic synthesis, conducted in five iterative steps (Thomas and Harden, 2008). First, each study was read in full and structured data were extracted: bibliographic details, design, sample characteristics (size, age, gender, educational stage, and country/cultural context), EI conceptualization and instrument, pedagogical model or intervention, SDT-related variables, key EI-related findings, and effect sizes where reported. Second, extracted data were coded into five predefined domains: EI theory and measurement; pedagogical models and teaching practices; SDT mechanism variables; EI development outcomes (proximal dimensions and distal adaptive outcomes); and cultural factors. Third, cross-domain relationships (e.g., “TGfU+SE → autonomy satisfaction → emotion-regulation improvement”) were identified and each graded for evidential strength on a four-level scheme (▲▲▲ = consistent evidence with meta-analytic support; ▲▲ = consistent evidence from two or more studies; ▲ = single-study or mixed evidence;? = insufficient or contradictory), with single-context findings flagged for cross-cultural verification. Fourth, an initial integrative framework—the Culturally Situated SDT–EI Model—was built from these relationships and then tested against the full corpus, with inconsistencies triggering revision until the model accommodated the body of evidence. Fifth, the model was cross-validated against the eight prior systematic reviews (Table 1) and each pathway assigned a transparent evidence grade, reported in the evidence-direction map (Table 3).

### 2.7 Effect direction synthesis

Given the heterogeneity of designs, instruments, and cultural contexts, a formal meta-analysis was neither feasible nor appropriate. To provide a structured, transparent, semi-quantitative synthesis, an effect direction plot was constructed (Thomson and Thomas, 2013). For each empirical study examining pedagogical model effects, the direction of effect on key EI dimensions—attention, clarity, regulation, empathy, and well-being—was coded as a significant positive effect (▲), a non-significant or mixed result (○), a significant negative effect (▼), or not measured (—); where a significant positive effect was recorded, the number of triangles denotes its magnitude by conventional benchmarks, as specified in the note to Table 4, and reported effect sizes (Cohen’s d, η^2^, or odds ratios) are annotated. This display convention is distinct from the four-level evidence-grading scheme described in Section 2.6 and applied in Table 3, which grades the strength of the evidence for a pathway across studies rather than the size of an effect within one; triangles in the two tables should therefore not be read as equivalent. This display summarizes the pattern of evidence while making its gaps visible: Outcomes a study did not measure appear as explicit absences rather than implied null effects.

**Table 4:** Effect direction plot: pedagogical model effects on EI dimensions.

Study	Model	Attention	Clarity	Regulation	Empathy	Well-being
Nazari et al. (2025)	TGfU+SE	—	—	▲▲	—	▲
Méndez-Giménez et al. (2022)	SEM	—	—	▲▲	▲	▲
Puente-Maxera et al. (2023)	SEM	—	—	▲	—	—
Rivera-Pérez et al. (2020)	Cooperative	▲▲	▲▲	▲▲▲	▲▲	—
Troncoso-Ulloa et al. (2025)	Mindfulness	▲	▲	○	—	—
Castillo Viera et al. (2021)	Dramatization	▲	—	▲	—	▲
Ortiz-Franco et al. (2024)	Judo	—	—	▲	—	▲

For the experimental and quasi-experimental studies, triangles denote standardized intervention-effect magnitudes (▲▲▲ = large, ▲▲ = medium, ▲ = small, by conventional Cohen benchmarks); ○ = non-significant; — = not measured. Rivera-Pérez et al. (2020) is the sole cross-sectional study; its triangles index strength of association (odds ratios), not intervention effects, and are therefore not directly comparable with the experimental rows—they should be read as a qualitative direction indicator only.

### 2.8 Theoretical lenses

Consistent with calls for transparency in narrative reviews, the analytic lenses are declared rather than assumed. The primary framework is self-determination theory (Ryan and Deci, 2000), specifically basic psychological need theory (Vansteenkiste et al., 2020). EI is analyzed through both the ability model (Salovey and Mayer, 1990; Mayer and Salovey, 1997) and the trait model (Petrides and Furnham, 2001), with each study’s operationalization recorded and the distinction treated as analytically consequential (see Section 3). The cultural lens is Markus and Kitayama’s (1991) independent–interdependent self-construal framework, informed by Cultural Sport Psychology’s view of culture as constitutive of psychological processes (Ryba, 2017; Schinke et al., 2019). Declaring these lenses does not imply that alternative interpretations are invalid; it enables readers to assess the boundary conditions within which the conclusions hold.

### 2.9 Methodological limitations

Five limitations of the method warrant note. First, and most consequentially for a review that advances a cross-cultural claim, the search was linguistically and bibliographically bounded. Searching in English and Spanish through six international databases systematically under-samples scholarship published in Chinese, Arabic, Portuguese, French, Russian, Hindi, and the many other languages in which PE research appears, and international indexes under-represent the regional journals that carry much Global South scholarship. The resulting corpus is correspondingly skewed: Roughly one-third of the evidence base originates in Spain; the cross-cultural layer of the model rests on four studies drawn from China, Russia, Ukraine, and four Arab states; Latin America is represented by a single Chilean intervention; and no study from sub-Saharan Africa, South Asia, or Southeast Asia contributes to the pedagogical or mechanistic evidence base. The implication is not merely that some studies were missed, but that the reach of the model is bounded. Its cultural moderation layer is evidenced for Western European, Eastern European, East Asian, and Arab educational contexts; for Latin America, sub-Saharan Africa, South Asia, and Southeast Asia, it is an untested extrapolation, and the model should be read accordingly. Two remedies follow. Analytically, the independent–interdependent contrast should be treated as the two poles for which evidence currently exists rather than as an exhaustive cultural taxonomy. Procedurally, any future update of this synthesis should be conducted as a multilingual search incorporating SciELO and Redalyc, CNKI and Wanfang, African Journals Online, J-STAGE and KCI, and South Asian regional indexes, with screening distributed across a linguistically diverse review team—the same recommendation this review makes to primary researchers in Section 7.3. Second, narrative subjectivity is inherent to thematic synthesis; explicit lensing and transparent grading manage rather than eliminate it. Third, publication bias may overrepresent positive findings, although the effect direction plot makes non-significant results visible. Fourth, the corpus’s near-uniform reliance on self-report EI instruments constrains what the synthesis can establish about the mechanism it proposes, as Section 3.2 develops in detail. Fifth, the cultural frameworks, while useful organizing heuristics, simplify complex realities—the within-region variability documented by Zayed et al. (2025) shows that even neighboring countries sharing language and religion may differ in how EI relates to well-being.

## 3 Theoretical foundations: defining and measuring EI in PE

### 3.1 Three paradigms of emotional intelligence

EI is not a unitary construct but a contested terrain comprising three conceptually distinct paradigms, each with different implications for PE research (O'Connor et al., 2019). The ability model (Salovey and Mayer, 1990; Mayer and Salovey, 1997) conceptualizes EI as a cognitive capacity to process emotional information, organized hierarchically: perceiving, using, understanding, and managing emotions. Measured through performance-based tasks such as the Mayer-Salovey-Caruso Emotional Intelligence Test (MSCEIT; Mayer et al., 2002), ability EI should increase with age and experience—predictions aligned with PE’s developmental objectives—yet ability-based measurement remains exceedingly rare in PE research. The trait EI model (Petrides and Furnham, 2001) defines EI as emotional self-efficacy—a constellation of self-perceptions located at lower levels of personality hierarchies, operationalized through instruments such as the Trait Emotional Intelligence Questionnaire (TEIQue; Petrides, 2009). Its dominance in PE research has enabled rapid evidence accumulation but constrains causal claims about whether PE genuinely develops emotional competence or merely alters self-perceptions. Mixed models (Bar-On, 2006; Goleman, 1995) combine cognitive abilities with personality traits and social skills into a broader composite, offering practical utility for intervention design at the cost of conceptual precision that complicates construct validation. Boyatzis’s (2018) behavioral level—how emotional competence appears in observable actions, assessed through multi-source feedback—provides a complementary perspective particularly salient for PE, where emotional competence is publicly displayed in teacher–student and peer interactions.

### 3.2 The measurement landscape: a critical audit

Three systematic biases emerge from the instrument landscape summarized in Table 5. First, self-report dominance: over 80% of studies employed self-report instruments—predominantly the Trait Meta-Mood Scale (TMMS-24) in Spanish/Latin American research and the Wong and Law Emotional Intelligence Scale (WLEIS) in East Asian research—with ability-based measures virtually absent. Without triangulation through performance-based or behavioral assessment, whether observed improvements represent genuine skill development, increased self-awareness, or response-shift bias remains ambiguous. Second, cultural asymmetry in instrument validation: instruments validated in one context are sometimes deployed cross-culturally without measurement invariance testing. Yahyaoui et al. (2024) demonstrated that their Arabic EI Scale (A-EIS) yielded a five-factor structure differing from both the WLEIS and TMMS-24, and Zayed et al. (2025) found scalar invariance did not hold across three Arab countries—invalidating direct cross-national mean comparisons. Third, a persistent theory-measurement mismatch exists: studies invoking the ability model’s theoretical framework frequently operationalize EI through self-report instruments, creating interpretive ambiguity about which paradigm’s predictions are being tested.

**Table 5:** EI measurement instruments in physical education and sport research.

Instrument	Paradigm	Dimensions	Prevalence	Cultural validation
TMMS-24	Trait	Attention, Clarity, Repair	Dominant (Spain/Latin America)	Spanish, English, Portuguese, Arabic, Turkish, Chinese
WLEIS	Trait	Self/Others’ Appraisal, Use, Regulation	Dominant (East Asia)	Chinese, English, Arabic, Russian, Korean
Schutte SSRI	Mixed/Trait	Perception, Management, Utilization	Moderate	English, multiple languages
TEIQue/TEIQue-SF	Trait EI	Well-being, Self-control, Emotionality, Sociability	Low (in PE research)	20 + languages
EQ-i / EQ-i: YV	Mixed	Intrapersonal, Interpersonal, Stress, Adaptability, Mood	Low (in PE research)	Multiple languages
MSCEIT	Ability	Perceiving, Using, Understanding, Managing	Very rare (in PE research)	English, Spanish
A-EIS	Trait	Five-factor Arabic-specific structure	Single validation study	Arabic: Tunisian PE students

These biases are not merely descriptive; they bear directly on what the SDT-based mechanism developed in Section 5 can be said to establish, and three implications warrant explicit statement. First, common method variance: the core evidence for the EI → need satisfaction link comes from studies in which EI, need satisfaction, and motivation were all measured by Likert-type self-reports completed by the same respondents in a single administration. Some portion of the observed association is therefore attributable to shared method rather than to shared construct, and the pathway grades in Table 3 should be read as upper bounds. Second, construct overlap: trait EI is defined as emotional self-efficacy—a structured set of self-perceptions—and perceived need satisfaction is similarly a perception of one’s own experience. A correlation between the two is thus partly definitional, and the inference that emotional competence produces need satisfaction cannot, on self-report data alone, be separated from the possibility that both index a common self-evaluative positivity. The null relatedness finding in Mercader-Rubio et al. (2022b) is open to the same reading: Relatedness is the one need whose satisfaction depends on others’ behavior rather than on self-appraisal and may therefore be the need least well captured by a self-appraisal instrument. Third, and most directly relevant to the ability paradigm, a study that invokes the four-branch ability model but operationalizes EI through the TMMS-24 or the WLEIS has not tested that model. Ability EI is defined as maximal performance on emotional-information-processing tasks and requires performance-based assessment scored against a criterion; self-report captures typical, self-perceived functioning, and the two correlate only modestly. Therefore, the ability model’s signature developmental prediction—that EI rises with age and experience—remains untested in PE, which is precisely the prediction most relevant to a review asking whether PE develops EI.

The consequence for the present synthesis should be stated without hedging. What the corpus supports is that need-supportive PE is associated with, and in six intervention studies followed by, improvement in students’ self-perceived emotional competence. Whether it develops emotional ability—the capacity to process emotional information accurately—or emotional self-concept is not resolvable from the available evidence, and the two are not interchangeable: a student who has become more confident about managing frustration has not thereby been shown to manage it more effectively. Behavioral evidence bears on this directly, and PE is unusually well placed to supply it because emotional competence in the gymnasium is publicly enacted rather than privately reported; Boyatzis’s (2018) behavioral level is therefore not merely a complementary perspective in this setting but the most feasible route to triangulation. Until intervention studies triangulate self-report with performance-based measurement (the MSCEIT or an equivalent) and systematic behavioral observation, the SDT-based account developed below should be understood as a mechanism specified at the level of perceived need satisfaction and perceived emotional competence, and its grades in Table 3 read in that light.

## 4 Pedagogical models as antecedents: how PE may nurture EI

### 4.1 Four core pedagogical models

#### 4.1.1 Teaching games for understanding + sport education (TGfU+SE)

The combined TGfU+SE model provides autonomy through tactical decision-making while SE provides role-based responsibility and sustained team affiliation. Nazari et al. (2025) provided proof-of-concept evidence: 72 Iranian secondary students in a 16-session football-based TGfU+SE intervention showed significant improvements in emotion regulation, intrapersonal intelligence, metacognition, and psychomotor skills compared to regular PE. However, this finding rests on a single study with no replication or follow-up, and no formal mediation analysis testing need satisfaction as the mechanism.

#### 4.1.2 Sport education model (SEM)

The SEM (Siedentop, 1994)—characterized by seasons, persistent team affiliation, formal competition, culminating events, and role differentiation—has accumulated the strongest evidence base. Méndez-Giménez et al. (2022) followed 252 Spanish secondary students across an entire school year with four measurement points, finding significant improvements in emotional control and regulation (η^2^ = 0.033) and emotional empathy (η^2^ = 0.007). Critically, SEM cushioned the motivational decline typically observed across the school year. Puente-Maxera et al. (2023) extended this evidence to a culturally diverse context (77 elementary students, 64% immigrant), demonstrating improvements across grades. However, both studies were conducted in Spain, and effect sizes were small, suggesting SEM alone may be insufficient without explicit emotional skills instruction.

#### 4.1.3 Cooperative learning and TPSR

Rivera-Pérez et al. (2020) provided the strongest correlational evidence in the literature: Among 1,332 Spanish students aged 10–20 years, students in high-cooperative learning PE classrooms were 3.79 times more likely to exhibit high emotional recognition and 4.78 times more likely to exhibit high emotional regulation and control. The Teaching Personal and Social Responsibility model (TPSR; Hellison, 2011)—progressing through five levels from respecting others’ rights to transferring responsibility outside the gymnasium—directly targets the empathy dimension of EI, although rigorous experimental evidence specific to its EI effects remains thinner than for SEM.

#### 4.1.4 Mindfulness, body expression, and emotional health PE

Troncoso-Ulloa et al. (2025) demonstrated that a 14-week emotional health PE program with 214 Chilean university students significantly improved emotional attention and clarity—but not emotional repair (the most complex regulatory dimension)—with small effect sizes (d ≈ 0.30). Castillo Viera et al. (2021) showed that an 8-week dramatization program in primary PE significantly improved emotional expressiveness and self-control, with particularly pronounced effects among male students. Ortiz-Franco et al. (2024) found that a 24-session judo intervention decreased correlations among violent behaviors and increased EI’s mitigating effect on aggressive attitudes—suggesting contact sports, when taught within structured pedagogical frameworks emphasizing respect and self-control, may strengthen the functional relationship between EI and behavioral regulation. Across all models measuring TMMS-24 dimensions, emotional repair consistently showed the smallest or non-significant improvements, consistent with the ability model’s prediction that active emotion regulation is the most developmentally advanced EI branch.

### 4.2 Cross-model comparison

Table 4 reveals four patterns. (1) The most robust effects appear in models that satisfy multiple needs simultaneously; even approaches operating primarily—though not exclusively—through a single need (cooperative learning and TPSR, which foreground relatedness via positive interdependence while also engaging competence through individual accountability) recruit more than one need in practice, consistent with SDT’s integrated need satisfaction thesis (Vansteenkiste et al., 2020). (2) The evidence base is culturally narrow: Every model has been tested primarily in Spain, with scattered single studies from Iran and Chile. No model has been replicated in East Asian, sub-Saharan African, South Asian, or Southeast Asian PE contexts, and none in Latin America beyond the single Chilean study. This is a substantive constraint rather than a gap to be noted in passing because each model presupposes conditions that are themselves culturally and materially variable: the Sport Education Model devolves authority to students, cooperative learning presumes classes small enough for genuine group interdependence, and TGfU presumes facility and equipment ratios that are far from universal. The replication program this implies is specified in Section 7.3. (3) Emotional repair is consistently the hardest dimension to move. (4) Evidence strength inversely correlates with cultural breadth—the model we are most confident about (SEM) has been tested in the narrowest cultural range.

### 4.3 The neglected antecedent: teacher EI and autonomy-supportive teaching

Granero-Gallegos et al. (2023) demonstrated that PE teachers’ autonomy support positively predicted students’ self-esteem through the mediation of students’ EI—positioning teacher behavior as an antecedent to student EI specifically. Leisterer and Paschold (2022) provided experimental evidence that high-autonomy-supportive PE teaching produced significantly greater positive affective valence (ηp^2^ = 0.13) and enjoyment (ηp^2^ = 0.17) than controlling teaching. The emotional labor demands on PE teachers—managing student behavior in publicly visible settings, navigating the physical and emotional exhaustion of the role—may be uniquely intense (Petiot et al., 2023; Richards et al., 2020). This convergence of evidence suggests that pedagogical models for developing student EI through PE are likely to be effective only to the extent that they are delivered by emotionally competent teachers in autonomy-supportive ways, placing teacher EI training—not merely curriculum design—at the center of the intervention logic.

The emotional labor construct specifies why this dependency holds. Emotional labor denotes the effort of producing professionally expected emotional displays (Hochschild, 1983), achieved either through surface acting—simulating an unfelt display—or through deep acting, in which the felt emotion is itself regulated to match what the situation requires (Grandey, 2000). The distinction matters because the two carry opposite costs: Surface acting is associated with exhaustion and depersonalization, whereas deep acting is more sustainable and amounts, in effect, to the public performance of the very emotion-regulation competence that PE sets out to teach. PE teaching intensifies both. Instruction takes place in large, acoustically difficult, physically expansive spaces in which the teacher’s own affect is continuously and unavoidably visible to an entire class at once; the subject matter generates competitive frustration, public failure, embodied self-consciousness, and interpersonal conflict with a frequency that classroom subjects do not; and the teacher carries simultaneous responsibility for physical safety. Richards et al. (2020) situate these demands organizationally, showing that PE teachers’ emotional labor is bound up with perceived organizational support, affective commitment, and job satisfaction—that is, that it is a condition produced by the school as much as a characteristic of the individual. Petiot et al. (2023), analyzing the career moments PE teachers in difficult contexts recall as most significant, find those moments to be overwhelmingly emotional in character. The marginal curricular status of PE in many school systems compounds the load since a teacher obliged to justify the legitimacy of the subject performs emotional labor toward colleagues and administrators as well as toward students.

This bears asymmetrically on the four pedagogical models in Table 6, and the asymmetry is instructive: The models that produce the strongest EI effects are precisely those that make the heaviest emotional-labor demands. A Sport Education season requires a teacher to devolve authority to student coaches, referees, and statisticians and then to tolerate the noise, error, and conflict that devolution produces—sustaining relational investment across months rather than lessons, and resisting the reassertion of control at exactly the moments when control feels most warranted. Cooperative learning obliges the teacher to adjudicate intra-group conflict and free-riding rather than to bypass them through individual tasks. TPSR positions the teacher as both model and arbiter of the responsibility levels students are asked to climb, which is deep acting by definition: A teacher who performs respect without feeling it is teaching the surface-acting script rather than the competence. Mindfulness and body-expression approaches require the teacher to be a credible regulator of their own arousal since the intervention’s content is in part the teacher’s demonstrated state. A depleted teacher does not simply implement these models less well; the failure mode is specific and predictable—reversion to the transmission-style, high-control default that is least emotionally costly to sustain. Vansteenkiste et al.’s (2020) distinction between need satisfaction and need frustration identifies the consequence: Controlling delivery does not merely fail to satisfy needs but actively frustrates them, so a structurally faithful implementation of SEM or TPSR by an exhausted teacher may leave students worse off than conventional PE would. This is the mechanism behind the observation that implementation quality, rather than model selection, is decisive, and it is consistent with evidence that sportive activity alone does not straightforwardly predict emotional intelligence competence (Tozoğlu and Erciş, 2025).

**Table 6:** Cross-model comparison of pedagogical approaches to EI development in PE.

Model	Primary SDT mechanism	Strongest EI outcome	Evidence	Cultural contexts tested
TGfU + SE	Autonomy + Competence + Relatedness	Emotion regulation; Intrapersonal intelligence	▲ (1 study)	Iran only
Sport Education	Autonomy + Competence + Relatedness	Emotional control/regulation; Empathy	▲▲ (2 studies, 1 longitudinal)	Spain; culturally diverse Spain
Cooperative Learning/TPSR	Relatedness + Competence (positive interdependence; individual accountability)	Emotional recognition; Regulation; Empathy	▲▲ (large cross-sectional)	Spain only
Mindfulness/Body Expression	Interoceptive awareness → Autonomy	Emotional attention; Clarity	▲ (small quasi-experimental)	Chile, Spain

Evidence column uses the four-level grading scheme defined in Section 2.6 (▲▲ = consistent evidence from two or more studies; ▲ = single-study or mixed evidence); study design in parentheses.

Two further specifications follow. First, the emotional-labor moderator is itself culturally calibrated. Zhang’s (2026) China–Ukraine comparison found that student satisfaction tracked teacher extraversion in China but teacher self-awareness in Ukraine and that Chinese teachers scored higher on self-management and authoritarian leadership while Ukrainian teachers scored higher on self-awareness, social awareness, and democratic leadership. The display rules that define emotionally competent PE teaching—how much warmth, how much distance, and how much emotional expression is legitimate from a teacher—are therefore locally specified, and an emotional-labor intervention designed against Western display norms may impose dissonance rather than relieve it elsewhere. Second, and candidly, the account offered here is a synthesis of adjacent literature rather than a tested proposition. Teacher EI and teacher emotional labor have been studied as predictors of teacher outcomes (Richards et al., 2020; Petiot et al., 2023), and teacher autonomy support has been studied as a predictor of student outcomes (Granero-Gallegos et al., 2023; Leisterer and Paschold, 2022), but no study in this corpus has measured teacher emotional labor and student EI within a single design, still less tested it as a moderator of a pedagogical model’s effect. Aponte et al. (2025) document the parallel gap in provision: effective educator EI programs exist, integrating SEL, mindfulness, emotional journaling, and virtual simulations, but none has been adapted to PE teacher education, whose demands differ from those of classroom teaching in exactly the respects set out above. The pathway is accordingly entered in Table 3 as untested, and its examination is added to the research agenda in Section 7.3.

## 5 SDT as the mechanistic core

### 5.1 The Mercader-Rubio research program

The research team led by Isabel Mercader-Rubio, Nieves Gutiérrez Ángel, and Sónia Brito-Costa has produced nine studies (2022–2023) constituting the most concentrated empirical investigation of the SDT–EI nexus in physically active populations. All employed cross-sectional designs with Spanish and Portuguese university sport science students (N ≈ 163–165), predominantly using the TMMS-24. Table 7 summarizes the core findings.

**Table 7:** Summary of the Mercader-Rubio research program on EI and SDT variables.

Study	Key SDT-related finding
Mercader-Rubio et al. (2022a)	EI significantly predicted identified, introjected, and external regulation
Mercader-Rubio et al. (2022b)	EI predicted autonomy and competence need satisfaction; relatedness was not significant
Mercader-Rubio et al. (2022c)	EI predicted motivation types, pre-competitive anxiety, and leadership qualities
Mercader-Rubio et al. (2022d)	EI predicted interpersonal relationships with gender as a mediating variable
Mercader-Rubio et al. (2022e)	EI dimensions predicted task-oriented and ego-oriented motivational orientations
Mercader-Rubio et al. (2023a)	EI related to intrinsic motivation to know, to achieve, and to experience stimulation
Mercader-Rubio et al. (2023b)	Emotional clarity and regulation correlated with self-confidence; negatively with somatic and cognitive anxiety
Mercader-Rubio et al. (2023c)	Seven psychological skill factors (self-confidence, coping, attentional control, etc.) related to EI dimensions
Mercader-Rubio et al. (2023d)	EI dimensions predicted informal sports leadership emergence

The most theoretically informative finding is the dissociation in Mercader-Rubio et al. (2022b): EI predicted autonomy and competence need satisfaction—but not relatedness. One reading is substantive: that EI, as an individual competence, more directly supports individual needs (autonomy through self-regulation; competence through effective emotional coping) than the inherently interpersonal need for relatedness. A second is methodological: Relatedness satisfaction depends on others’ responses rather than on self-appraisal and may be the need least well captured by the self-report instruments used throughout this program (Section 3.2). The two readings cannot be distinguished within a cross-sectional, single-method design, and the finding rests on one sample of approximately 165 students. Should it be replicated across cultures, age groups, and measurement approaches, the substantive reading would carry a practical implication: PE programs targeting EI through the relatedness pathway (cooperative learning, TPSR) may need to supplement social-structural intervention with explicit emotional skills instruction. Because the entire program is cross-sectional, the directional vocabulary used throughout this section—predicted, related to—should be read as statistical prediction within a correlational design rather than as evidence of causal precedence.

### 5.2 Cross-validation and evidence grading

Öztürk Çelik (2025) demonstrated that trait EI predicted psychological well-being among 226 Turkish PE athlete-students, partially mediated by self-efficacy (a competence-related construct within SDT) and burnout (the endpoint of need frustration). Vasconcellos et al. (2020) meta-analyzed 265 independent samples, confirming that need-supportive teaching practices robustly predict autonomous motivation (*ρ* = 0.35–0.45), which in turn predicts adaptive outcomes including skill development and psychological well-being. Theodosiou et al. (2021) concluded that the SDT motivational sequence—autonomy support → need satisfaction → well-being—is largely invariant across Greek and UK PE students, providing necessary (though not sufficient) support for SDT’s cross-cultural applicability. Table 3 synthesizes the evidence for each pathway using the transparent four-level grading scheme.

Vansteenkiste et al. (2020) provide three conceptual refinements from the BPNT literature that sharpen the mechanistic account. (1) Need satisfaction versus need frustration—a controlling teaching style may not merely fail to develop EI but actively undermine it, by teaching students that their emotions are not valid objects of attention or regulation. This explains why implementation quality, not merely model selection, is critical. (2) The universality-with-variability thesis—basic psychological needs are posited as universal, but the means of satisfying them, and the relative weighting of different needs, vary across cultural contexts. This provides the theoretical foundation for the cross-cultural analysis in Section 6. (3) The physical-psychological need interface—physical fatigue, pain, and bodily self-consciousness in PE can facilitate or impede emotional self-regulation, a dual role of the body as simultaneously vehicle of need satisfaction and potential source of need frustration that is insufficiently theorized in current PE–EI research.

### 5.3 The causal status of the evidence

The mechanistic account assembled above is a chain of associations, and its causal reading should be advanced with corresponding restraint. Three constraints define what may and may not be claimed. First, direction: with the exception of Vasconcellos et al.’s (2020) meta-analysis of need-supportive teaching, every study linking EI to SDT variables in this corpus is cross-sectional, and the reverse pathway is theoretically at least as plausible as the forward one—students who experience their needs as satisfied are better placed to attend to, clarify, and regulate emotion than students who do not. Reciprocal causation, in which need satisfaction and emotional competence reinforce one another across a school year, is the most likely account of all and the one the available designs are least able to detect. Second, mediation: no study in Table 4 measured need satisfaction and EI at multiple time points within an intervention and tested the indirect effect. The pedagogical model → need satisfaction → EI pathway is therefore inferred from parallel improvements in variables measured in separate studies, which is why it carries one of the lowest grades in Table 3 despite being the model’s central claim. Third, confounding: intervention studies in PE are rarely blinded and seldom control for teacher enthusiasm, novelty, or expectancy, so effects attributed to a model’s structural features may partly reflect the attention and energy that accompany implementing something new. Méndez-Giménez et al.’s (2022) year-long, four-wave design and Rivera-Pérez et al.’s (2020) large cross-stage sample are the strongest evidence available, and neither licenses a causal claim on its own: The former is a single-country study without a formal mediation test, while the latter is a cross-sectional association reported as odds ratios. The claims advanced in Section 7 are accordingly framed as propositions the evidence renders plausible and coherent rather than as established causal relations; where the phrasing of this review implies a directional process, it describes the hypothesized mechanism under test, not a demonstrated one.

## 6 Cross-cultural perspectives: the cultural moderation of EI mechanisms

### 6.1 Why culture must enter the model

Three converging considerations motivate culture’s elevation from a limitation paragraph to a formal model component. First, the WEIRD problem: approximately 99% of sport and exercise psychology research is authored from Western universities (Cormack and Hand, 2022). Of the studies forming this review’s evidence base, roughly one-third originate in Spain, with Chinese, Turkish, Iranian, Chilean, and Arab-region samples contributing much of the remainder, and no study from sub-Saharan Africa, South Asia, or Southeast Asia contributing to the pedagogical or mechanistic evidence base—a systematic exclusion of populations whose cultural models of self, emotion, and education may differ fundamentally from the WEIRD default, and one this review reproduces rather than corrects (Section 2.9). Second, Markus and Kitayama (1991) distinguished between the independent self-construal (predominant in Western cultures; the self as bounded and autonomous; emotional authenticity and self-expression valued) and the interdependent self-construal (predominant in many East Asian cultures; the self as fundamentally connected to others; emotional harmony and appropriate restraint valued). In independent contexts, EI manifests as the capacity to recognize and assert one’s own emotions. In interdependent contexts, EI manifests as the capacity to perceive others’ emotions and regulate one’s own to maintain group harmony. Third, Cultural Sport Psychology (Schinke et al., 2019; Ryba, 2017) has established that sport and PE are cultural practices, not culturally neutral activities. From a CSP perspective, the SDT–EI model developed here is itself a cultural product—formulated by Western-trained researchers using constructs (autonomy and intrinsic motivation) carrying specific Western cultural meanings. The question is not whether SDT applies outside WEIRD contexts but how its application must be culturally nuanced.

### 6.2 Three direct cross-cultural comparisons and a cultural validation

#### 6.2.1 China vs. Russia

Rogaleva et al. (2024) compared 474 student-athletes from PE universities on EI (WLEIS), autonomous motivation, and physical activity. Chinese students scored significantly higher on all four WLEIS dimensions—self-emotion appraisal, others’ emotion appraisal, use of emotion, and emotion regulation—challenging the assumption that EI is higher in individualistic cultures. Critically, the dimensions predicting autonomous motivation differed: self-appraisal and regulation in China; only use of emotion in Russia. EI explained 42.6% of autonomous motivation variance among Chinese students versus a substantially lower proportion among Russian students. The authors attributed these differences to sport cultural traditions: Eastern psychophysical integration versus Western competitive instrumentality.

#### 6.2.2 China vs. Ukraine

Zhang (2026) compared 600 students and 20 PE teachers. Ukrainian teachers scored higher on self-awareness, social awareness, and democratic leadership; Chinese teachers excelled in self-management and authoritarian leadership. The correlates of student satisfaction differed by country—associated with teacher extraversion in China (r = 0.164) but teacher self-awareness in Ukraine (r = 0.431)—demonstrating that what students perceive as emotionally competent teaching is itself culturally shaped.

#### 6.2.3 Three Arab countries

Zayed et al. (2025) tested the EI → well-being → psychological distress pathway in 788 PE undergraduates from Oman, Kuwait, and Jordan. Most consequentially, well-being significantly mediated the EI → reduced distress pathway in Kuwait but not in Oman or Jordan. Scalar invariance did not hold across the three countries. This within-region dissociation refutes the assumption that broad cultural-geographic categories function as homogeneous blocs, demanding finer-grained cultural specification.

#### 6.2.4 Arabic scale validation

Yahyaoui et al. (2024) developed and validated the A-EIS with 651 Tunisian PE students. The confirmatory factor analysis yielded a five-factor structure—appraisal of others’ emotions, appraisal of own emotions, regulation, social skills, and utilization of emotions—differing from both the four-factor WLEIS and three-factor TMMS-24 structures, suggesting that the dimensionality of EI itself may be partly culturally constituted, addressing a measurement gap affecting approximately 400 million Arabic speakers worldwide.

### 6.3 Cultural modulation across model layers

The evidence motivates three modifications to the SDT–EI model, each a hypothesis generated by the cross-cultural comparisons rather than a finding established by them. At the antecedent layer, no pedagogical model in Table 6 has been independently replicated outside European and Middle Eastern contexts, the single Chilean study excepted. In high power distance cultures, SEM’s emphasis on student autonomy may require scaffolded introduction with initial teacher-led structure; in collectivist cultures, cooperative learning and TPSR may be particularly culturally congruent and, consequently, more effective. At the mechanism layer, the ‘China paradox’—higher EI in a higher power distance culture—resolves if autonomy is recognized as culturally construed: In interdependent contexts, aligning one’s behavior with authoritative guidance and group norms may be experienced as autonomous, an expression of the interdependent self rather than its suppression. This interpretation is supported by Shen et al. (2022), who found that students with collectivist beliefs perceived authority support as autonomy-supportive. At the outcome layer, the EI dimensions most valued in a given culture, and therefore most readily developed, differ. This does not imply that certain dimensions should be neglected but that PE–EI programs should identify and build upon culturally valued dimensions before extending to less culturally emphasized competencies. None of the three modifications has been tested directly: The comparisons in Table 8 were conducted on EI and motivation variables rather than on pedagogical model effects, so the antecedent-layer claims in particular are extrapolations awaiting the replication program set out in Section 7.3.

**Table 8:** Cross-cultural studies of EI-related variables in PE and sport.

Study/comparison	Sample	Key cultural finding	Implication for SDT–EI model
China vs. Russia (Rogaleva et al., 2024)	474 student-athletes	EI dimensions driving motivation are culture-specific	Universal structure, culturally variable expression pathways
China vs. Ukraine (Zhang, 2026)	600 students + 20 PE teachers	Effective teacher EI profile is culturally contingent	Teacher EI training must be culturally calibrated
Oman vs. Kuwait vs. Jordan (Zayed et al., 2025)	788 PE undergraduates	EI–well-being pathways differ even within a single regional bloc	Cultural moderation operates at sub-regional granularity
Tunisia (Yahyaoui et al., 2024)	651 PE students	Five-factor EI structure differs from Western models	EI dimensionality may be partly culturally constituted

## 7 Discussion

### 7.1 The culturally situated SDT–EI model of physical education

**Figure 1:**
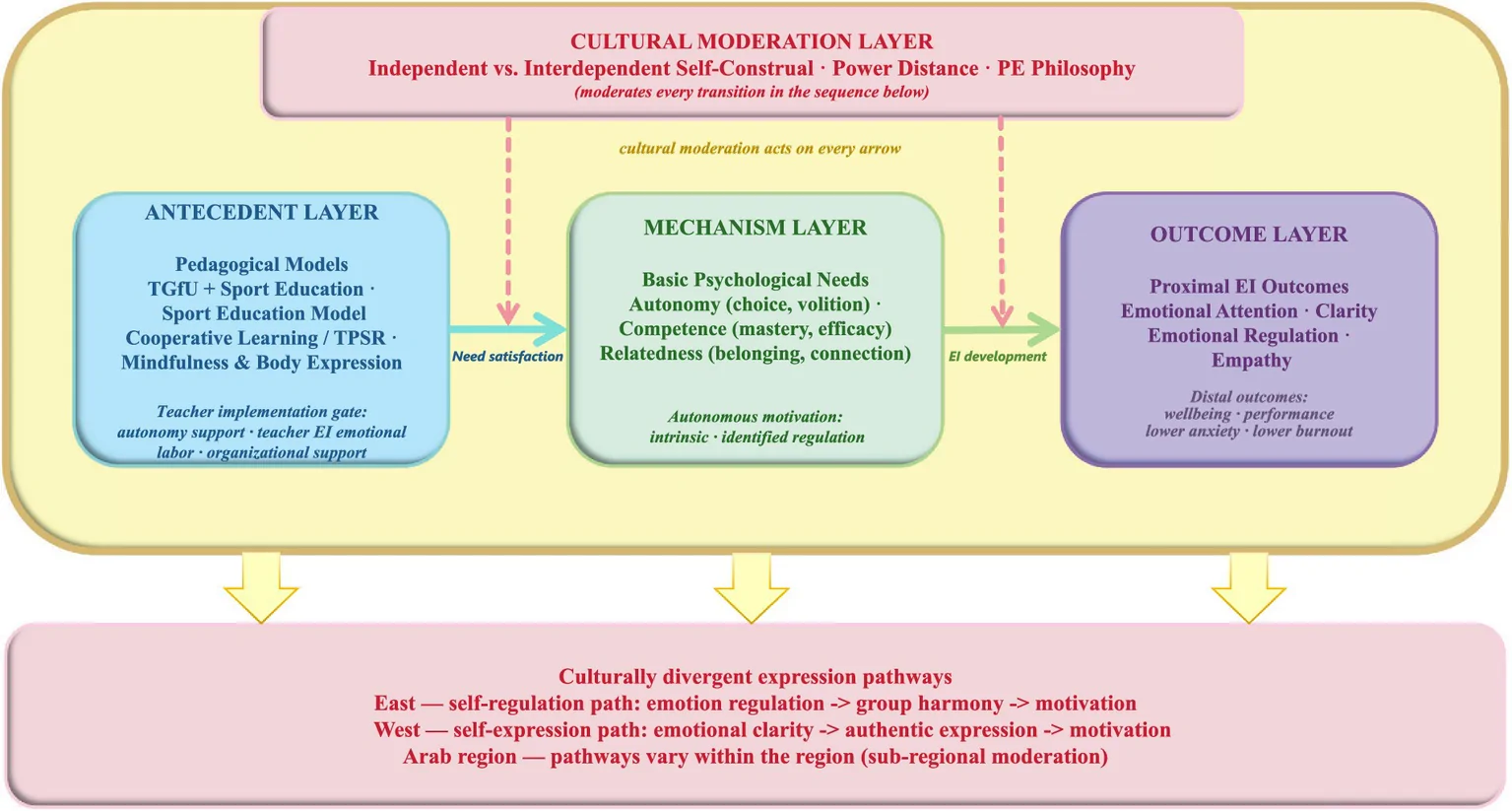
The proposed Culturally Situated SDT–EI Model of Physical Education. The model is derived from narrative synthesis of the literature reviewed here and has not been tested as an integrated structure; the evidence grade for each pathway is reported in Table 3.

Synthesizing the preceding sections, we propose the Culturally Situated SDT–EI Model of Physical Education (Figure 1). The model posits that PE develops EI when—and to the extent that—pedagogical models create conditions that satisfy students’ basic psychological needs for autonomy, competence, and relatedness. Need satisfaction is held to enable the internalization of autonomous motivation, which in turn is held to support the development of emotional competencies (attention, clarity, regulation, and empathy), yielding distal adaptive outcomes including psychological well-being, academic performance, reduced anxiety, and reduced burnout. Teacher emotional competence and the emotional labor conditions under which teaching is carried out enter the model at the antecedent layer as a gating condition on implementation (Section 4.3). This sequence—antecedent conditions → need satisfaction → autonomous motivation → EI development → distal outcomes—is proposed to be universal in structure but culturally variable in operationalization: In independent-self educational cultures, EI development is expected to follow a self-expression pathway, and in interdependent-self educational cultures, a self-regulation pathway.

The epistemic status of the model requires explicit statement. It is a synthesis-derived proposition, not an empirically validated structure. No study has yet measured its antecedent, mechanism, moderator, and outcome layers within a single design, and several of its links—the mediation of pedagogical effects by need satisfaction, the moderating role of teacher emotional labor, and the cultural specification of the antecedent layer—rest on inference across studies rather than on direct test (Table 3). Therefore, the model is offered as a structured set of falsifiable hypotheses whose value lies in the research it makes possible and should be cited as such rather than as a validated framework. Validating it would require, at minimum, a multi-site longitudinal study in which a single pedagogical model is implemented in at least three culturally distinct educational systems, with need satisfaction, motivation quality, and EI measured at three or more waves, EI assessed by more than one method, measurement invariance of every instrument established across sites before any cross-site comparison is attempted, and the moderating hypotheses tested through multi-group structural equation modeling. Until such evidence exists, the model organizes the literature and generates predictions; it does not certify them.

Five principles govern the model. (1) Layered causality: EI development requires aligned conditions across the antecedent, mechanism, and moderation layers; a failure at any layer can break the chain, which would explain why unstructured sport participation alone does not reliably develop EI (Tozoğlu and Erciş, 2025). (2) Universal structure, culturally variable expression: needs are posited as universal, but the practices that satisfy them—and even the meaning of ‘autonomy’ itself—vary across cultural contexts. (3) Differential EI development: different EI dimensions are proposed to develop through partly distinct pathways, with emotional repair consistently the hardest to move—a level of specificity not foregrounded in prior reviews. (4) Implementation dependency: the antecedent layer is gated by the teacher so that model effects are conditional on delivery by an emotionally competent teacher working under organizational conditions that make deep rather than surface acting sustainable; the same model may therefore produce need satisfaction or need frustration depending on who delivers it and under what support (Section 4.3). (5) Evidence-graded confidence: each pathway is transparently coded (Table 3), making the model falsifiable rather than dogmatic.

### 7.2 Theoretical and practical contributions

The model is intended to make four theoretical contributions to the existing literature, each a claim about the framework’s organizing value rather than about validated findings. First, it integrates pedagogical antecedents, SDT mechanisms, EI outcomes, and cultural moderation within a single framework spanning preschool to university—an integration not undertaken by the eight prior reviews considered individually. Second, culture is elevated from a demographic descriptor to a formal model component generating specific, falsifiable predictions (e.g., SEM will produce smaller EI gains in high power distance PE cultures unless adapted to incorporate initial teacher-led structure). Third, transparent evidence grading makes explicit what is claimed with confidence versus provisionally—an important corrective in a field where causal claims about EI development often outrun the cross-sectional evidence supporting them. Fourth, the specification of differential EI development pathways—and the prediction that emotional repair is the hardest dimension to move—offers a specific, testable refinement. Whether these contributions hold is itself an empirical question, and each is stated in a form that permits it to be answered negatively.

Practically, the model generates three design principles for culturally responsive PE–EI programs. (1) Structure PE for multi-need satisfaction—simultaneously supporting autonomy (genuine choices in movement engagement, role assumption, and emotional reflection), competence (optimally challenging tasks requiring genuine emotional engagement and coping with failure), and relatedness (persistent group structures enabling trust and mutual emotional support across multiple sessions). (2) Calibrate EI targets to cultural context—identifying and building upon the EI dimensions most valued in the specific cultural setting before extending to less culturally emphasized competencies. In interdependent-self cultures, programs might begin with emotion regulation and others’ emotion appraisal before introducing individual emotional self-expression; in independent-self cultures, programs might begin with emotional attention and clarity before extending to interpersonal dimensions. This is not cultural relativism but a developmental strategy that begins with cultural strengths. (3) Embed teacher EI development within program implementation—a Sport Education season delivered by an emotionally exhausted, controlling teacher is unlikely to develop student EI regardless of how faithfully its structural features are implemented. PE–EI programs must include pre-implementation teacher EI training targeting teachers’ own emotional competencies, ongoing peer coaching and reflective supervision that acknowledges the unique emotional labor demands of PE teaching, and cultural reflexivity training enabling teachers to recognize how their own cultural assumptions about emotion, authority, and the teacher–student relationship shape their pedagogical practice. These are design recommendations derived from the model rather than conclusions from trials of the principles themselves; they are offered as hypotheses for implementation research, and programs built on them should be evaluated rather than assumed effective.

### 7.3 Limitations and research agenda

The model’s boundary conditions define an eight-priority research agenda. (1) Longitudinal studies tracking the full SDT–EI pathway across multiple school years, with need satisfaction, motivation quality, and EI measured at each time point. (2) Formal mediation analyses within experimental designs, establishing need satisfaction as the causal mechanism linking pedagogical models to EI development, with the mediator measured during rather than only after the intervention. (3) Cross-cultural replication of the pedagogical models beyond the Iberian evidence base. Concretely, the Sport Education Model, as the model with the strongest evidence, should be replicated as a season of comparable length and structure in at least one East Asian setting (China, Japan, or Korea), one South or Southeast Asian setting (India, Pakistan, Bangladesh, Indonesia, or the Philippines), one sub-Saharan African setting (Nigeria, Kenya, Ghana, or South Africa), and one Latin American setting beyond Chile—ideally as a coordinated multi-site study with a shared protocol, pre-registered hypotheses, locally validated EI instruments, and measurement invariance established before any cross-site comparison is attempted. Such replication should test the model’s structure and not merely its effect: whether the same need-satisfaction pathway obtains, and whether SEM’s devolution of authority requires the scaffolded, initially teacher-led introduction the model predicts for high power distance settings. Cooperative learning and TPSR warrant parallel replication in collectivist settings, where the model predicts they should perform comparatively well. (4) Multi-method EI assessment triangulating self-report with ability-based measurement (MSCEIT) and systematic behavioral observation in intervention studies, without which the field cannot distinguish developed emotional ability from raised emotional self-concept. (5) Teacher EI experimentally manipulated or measured as an independent variable in PE–EI studies—currently untested. (6) Teacher emotional labor measured alongside student outcomes within the same design, testing surface versus deep acting as a moderator of pedagogical model effectiveness, together with development and evaluation of a PE-specific adaptation of the educator EI programs whose absence Aponte et al. (2025) document. (7) Dimension-targeted interventions testing the model’s prediction that different EI dimensions develop through distinct SDT-mediated pathways, with emotional repair as the critical test case. (8) A direct test of the integrated model as specified in Section 7.1, accompanied by a multilingual update of the evidence base incorporating regional databases, so that the cultural layer is built on scholarship produced in the regions it purports to describe rather than on Western-indexed research about them.

The most important limitation is the status of the model itself: It is a product of synthesis, has not been tested as an integrated structure, and may require substantial revision—or abandonment—once it is. Methodologically, the core evidence for the SDT–EI pathway is predominantly cross-sectional, precluding definitive causal inference about the direction of the EI ↔ need satisfaction relationship (Section 5.3). Over 80% of reviewed studies used self-report EI measures, so the mechanism is specified at the level of perceived rather than demonstrated competence (Section 3.2). The bibliometric component was an exploratory scan that informed thematic organization rather than a formal co-occurrence or cluster analysis, which lay beyond the scope of this narrative synthesis and is therefore not reported as a stand-alone bibliometric result. The pedagogical models reviewed in Section 4 have been tested primarily in Spain, with scattered single studies from Iran and Chile; the evidence base is further bounded by language and database coverage in ways that fall most heavily on the Global South (Section 2.9), so the cross-cultural claims that most distinguish this review also rest on its narrowest evidential base. The SDT-centric theoretical lens is a choice, not a necessity: alternative frameworks—Social cognitive theory (Bandura, 1986), Gross’s process model of emotion regulation (Gross, 2015), and Fredrickson’s broaden-and-build theory (Fredrickson, 2001)—could organize the same evidence differently and generate different integrative insights. The East–West cultural comparison employed broad categories (independent vs. interdependent self-construal) that the Zayed et al. (2025) within-region finding demonstrates require finer-grained specification. The model’s scope is bounded by the PE context: Its claims may not generalize to other EI-development settings (classroom SEL, clinical intervention, and workplace training).

## 8 Conclusion

The evidence reviewed here is most coherently organized by the proposition that physical education develops emotional intelligence through a sequence of need-satisfying experiences that enable the internalization of autonomous motivation—and by the further proposition that this sequence is not culturally invariant. In independent-self educational cultures, EI development appears to follow a self-expression pathway: autonomy understood as individual choice, competence as self-referenced improvement, and emotional authenticity as the developmental goal. In interdependent-self educational cultures, EI development appears to follow a self-regulation pathway: Autonomy understood as volitional alignment with group-valued goals, competence as socially recognized contribution, and emotional harmony as the developmental goal. These are best understood not as competing accounts but as culturally modulated expressions of what may be a universal human process—a reading the present evidence supports but does not establish. Both propositions remain well-founded hypotheses rather than settled conclusions: They rest on a literature that is predominantly cross-sectional, almost entirely self-report, and linguistically and geographically bounded, and the model that assembles them awaits the direct empirical test set out above. Physical education nonetheless reaches nearly every child in every country. If it is designed—through evidence-based pedagogical models, implemented by emotionally competent teachers, calibrated to cultural context—to develop emotional intelligence alongside physical fitness, its contribution to human development may extend far beyond the gymnasium. This review has attempted to provide the conceptual architecture for that design, and the research agenda by which it can be put to the test.
